# Comparison of Anterior Segment Measurements Obtained by Three Different Devices in Healthy Eyes

**DOI:** 10.1155/2014/498080

**Published:** 2014-06-02

**Authors:** Carmen Lopez de la Fuente, Ana Sanchez-Cano, Francisco Segura, Isabel Pinilla

**Affiliations:** ^1^Department of Applied Physics, University of Zaragoza, C/Pedro Cerbuna 12, 50009 Zaragoza, Spain; ^2^Aragon Health Sciences Institute, Avenida San Juan Bosco 13, 50009 Zaragoza, Spain; ^3^Department of Surgery, Gynecology and Obstetrics, University of Zaragoza, C/Pedro Cerbuna 12, 50009 Zaragoza, Spain; ^4^Department of Ophthalmology, Lozano Blesa University Hospital, Avenida San Juan Bosco 15, 50009 Zaragoza, Spain

## Abstract

*Purpose*. To assess the normal values and the repeatability of the Galilei Dual Scheimpflug Analyzer (GDSA), the biometer IOL Master, and the autokerato/refractometer WAM 5500 in anterior segment examinations. *Methods*. Eighty-eight eyes from 88 healthy volunteers were prospectively and consecutively recruited. The repeatability was assessed, calculating the intraclass correlation coefficient (ICC). *Results*. The correlations among the repeated measurements showed nearly perfect reliability (ICC > 0.81) for all of the parameters, except corneal astigmatism Galilei (0.79) and WAM (0.68). There were statistically significant differences (*P* < 0.001) between the values of the flat simulated keratometry (SimK) and the steep SimK measured by GDSA and the other methods; however, there were no statistically significant differences for the values obtained with the IOL Master and WAM 5500 (*P* = 0.302 and *P* = 0.172, resp.) or between the values of the ACD (*P* < 0.001) and WTW (*P* = 0.007) measured by the IOL Master and GDSA. *Conclusions*. The anterior segment measurements from the IOL Master and WAM 5500 were highly repeatable, comparable, and well correlated. In healthy young persons, the evaluated parameters had very good repeatability, although significant differences were found between the GDSA and IOL Master and between the GDSA and WAM 5500.

## 1. Introduction

Accurately measuring the structures of the anterior segment is important for diagnosing diverse pathologies and for cataract, glaucoma, refractive surgery, and postsurgical control. The values of the anterior chamber depth (ACD), corneal power, and corneal astigmatism are essential in calculating the intraocular lens (IOL) with the newer theoretical biometric formulas. The measurement of the ACD is critical to the success of phakic IOL implantation in refractive surgery. Errors in the measurement of these parameters before surgery might result in postoperative refractive errors. Reducing postoperative refraction depends upon correct preoperative evaluation of the anterior segment measurements [1, 2].

Different technologies are used in the characterisation of the structures of the anterior segment. Typically, autokerato/refractometers, such as the WAM 5500 (Grand Seiko Co., Ltd., Hiroshima, Japan), are used; however, noncontact methods of ACD measurement have become preferred because of their speed, relative ease of use, avoidance of topical anaesthesia, and lack of corneal indentation. In recent years, techniques of anterior segment imaging have developed rapidly; new devices based on Scheimpflug images, such as the Pentacam (Oculus, Wetzlar, Germany) and Galilei Dual Scheimpflug Analyzer (GDSA) (Ziemer Group, Port, Switzerland), or high-speed anterior segment optical coherence tomography, such as the Visante (Carl Zeiss Meditec, Inc., Dublin, CA, USA), have been combined with previously established techniques, including biometrics based on the partial coherence interferometry (IOL Master, Zeiss, Jena, Germany), slit scanning (Orbscan, Bausch & Lomb, Rochester, New York, USA), or very high frequency ultrasound (Artemis, Ultralink, LLC, St. Petersburg, FL, USA). These devices provide qualitative and quantitative information regarding the cornea and anterior chamber and have the advantage of being noncontact techniques with ease of handling [3–8].

This study aims to compare different measures of healthy anterior segment structures obtained with three instruments and to determine whether these results could be used interchangeably in clinical practice. For this purpose, corneal measurements (keratometry, corneal power, and amount of astigmatism) from the Galilei, IOL Master, and WAM 5500 techniques were compared. Additionally, the anterior chamber measurements and the corneal diameter obtained with Galilei and IOL Master were compared.

## 2. Materials and Methods

Eighty-eight eyes from 88 healthy volunteers were prospectively and consecutively recruited from February 2012 to June 2013. The subjects were recruited from the University of Zaragoza; all of the subjects were students of the Optics and Optometry Degree program. A complete optometric examination was performed to exclude any ocular pathology. The prospective study protocol was approved by the Clinical Research Ethics Committee of Aragón (CEICA), and the volunteers provided written informed consent before inclusion in the study. The study design followed the standards of the Declaration of Helsinki for biomedical research.

Patients with ocular disease, previous ocular surgery, and systemic diseases with ocular implications were excluded. One eye was randomly selected from each subject. The parameters analysed were the anterior flat and steep simulated keratometry (SimK) parameters, anterior corneal astigmatism, ACD, and the white-to-white distance (WTW).

### 2.1. Autokerato/Refractometer WAM 5500

The autokerato/refractometer WAM 5500 is an open-field binocular device. Its open view condition allows a more natural measurement of the refraction, minimising instrumental myopia. This equipment allows for simultaneous obtainment of the refraction and keratometry values. While the patient focused on a nonaccommodative stimulus placed 6 m from the evaluated eye, three measurements of refractive error and corneal curvature were performed by an experienced optometrist [5, 9].

### 2.2. IOL Master

The IOL Master is a rapid and accurate noncontact biometry system. This device uses partial coherence interferometry (PCI) technology for the axial length measurements. This device is able to simultaneously perform keratometry and measurements of the ACD and WTW. The ACD measurement is performed using the slit-image technique, and software calculates the measurement between the anterior surface of the cornea and the anterior surface of the lens. For corneal curvature, six points of light are projected on the cornea in a hexagonal pattern. The instrument measures the distances between opposite points, securing three meridians, and calculates the corneal curvature [3, 10–13]. Five consecutive measurements of the corneal curvature, ACD, and WTW were obtained.

### 2.3. Galilei

The Galilei Dual Scheimpflug Analyzer employs two examination techniques for the analysis of the anterior segment. This device combines Placido rings with photography with a dual Scheimpflug camera to obtain more accurate measurements and a three-dimensional reconstruction of the anterior segment. During the scanning process, the G2 system (software version 5.2.1) acquires a series of 15/2 Scheimpflug 3D images and two Placido images with 90 degrees of separation; the system analyses 122.000 points of the anterior segment of the eye. The data obtained from the two camera positions are averaged to compensate for involuntary misalignment. The ACD measurement is taken from the endothelium to the anterior surface of the lens. The boundaries of the anterior cornea, posterior cornea, lens, and iris are detected by Scheimpflug imaging [10, 14, 15]. Three measurements of the anterior flat SimK and steep SimK parameters, anterior corneal astigmatism, ACD, and WTW were taken with this device. The Galilei system measures the ACD from the endothelium to the anterior surface of the lens; the IOL Master measures the ACD from the anterior corneal surface to the anterior lens surface. The central corneal thickness (CCT) obtained with the Galilei was added to the ACD, with the aim of comparing the two measures. The ACD acquired with the Galilei was designated as ACD_G_, and the ACD_IOL_ was obtained with the IOL Master.

## 3. Statistical Analyses

The statistical analyses were performed using the Statistical Package for the Social Sciences (SPSS 13.0, SPSS, Inc., Chicago, IL, USA). To assess the reliability of the repeated measurements with the devices, intraclass correlation coefficients (ICC) were used. The ICC is defined as the ratio of the between-subjects variance to the sum of the pooled within-subjects variance (*S*_*w*_) and the between-subjects variance. The ICC interpretation that was used considered the reliability of the values as follows: slight reliability for values between 0 and 0.2, fair reliability for values from 0.21 to 0.4, moderate reliability for values between 0.41 and 0.6, substantial reliability for values from 0.61 to 0.8, and almost perfect reliability for values higher than 0.81. The coefficient of variation (COV) was calculated as the* S*_*w*_ divided by the mean of the measurements and was expressed as a percentage; a lower COV indicates higher repeatability.

For the evaluation of the anterior segment measurements, the values of flat SimK, steep SimK, corneal astigmatism, ACD, and WTW obtained with the IOL Master, Galilei, and WAM 5500 were compared by paired Wilcoxon tests. Values of *P* < 0.05 were considered to be statistically significant differences. Martin Bland and Altman plots were used to assess agreement; all of the procedures were represented by displaying the differences between the measurements of the two methods against the mean of the two measurements [16, 17].

## 4. Results

Of the 88 patients assessed, there were 60 females and 28 males. The mean age was 22.30 ± 2.15 years.

Table 1 shows the mean flat SimK, steep SimK, corneal astigmatism, ACD, WTW, and repeatability findings for each device. The majority of the measured parameters were highly repeatable, with ICCs being higher than 0.93, with the exception of the corneal astigmatism measured by the Galilei (0.855) and WAM 5500 (0.790). The highest ICC was obtained using the flat SimK and steep SimK taken with the IOL Master, achieving levels of 0.999. All the COVs were below 4%, except that of the Galilei ACD (6.50%) and the IOL Master ACD (8.23%). The exceptions were corneal astigmatism (IOL Master: 55.35%, Galilei: 53.54%, and WAM 5500: 58.78%); the extremely high COVs of these measurements result from the proximity to zero of the mean value of the parameters and the sensitivity of the values to changes; these values could not be considered relevant.

The results of the flat SimK, steep SimK, and corneal astigmatism measured by the IOL Master, Galilei, and WAM 5500 were compared in pairs by Wilcoxon tests; the values of the ACD and WTW obtained with the IOL Master and Galilei were too compared in pairs by Wilcoxon tests. Statistically significant differences (*P* < 0.001) were found between the values of the flat SimK and steep SimK measured by the Galilei and the other methods; there were no statistically significant differences for the values obtained with the IOL Master and the WAM 5500 (*P* = 0.302 and *P* = 0.172, resp.). There were statistically significant differences for the values of corneal astigmatism measured using the Galilei and WAM 5500 (*P* = 0.022). There were statistically significant differences between the values of the ACD (*P* < 0.001) and WTW (*P* = 0.007) measured by the IOL Master and Galilei (Table 2). Despite these results, the measurements between the devices were well correlated; see Table 3.

**Figure 1:**
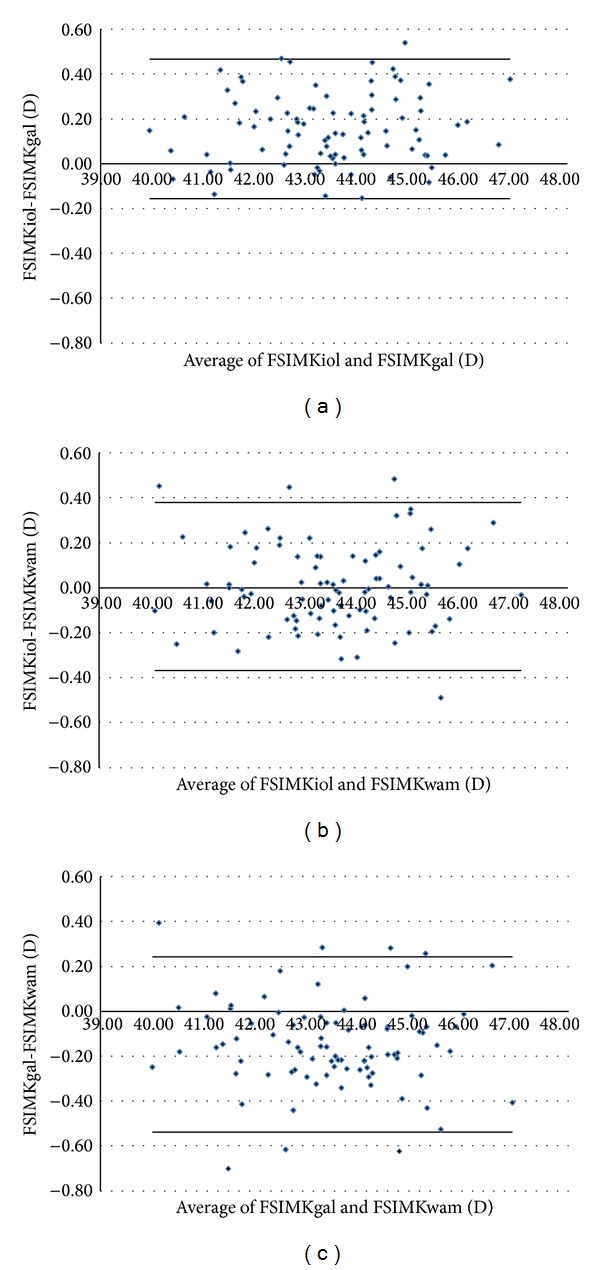
Bland-Altman analysis showing the distribution of the flat SimK differences on the *y*-axis and the average of the instrument readings on the *x*-axis. The IOL Master and Galilei, above (mean Δ ± SD: +0.155 ± 0.156 D, with 95% limits of agreement between −0.157 and +0.467); IOL Master and WAM 5500, middle (mean Δ ± SD: +0.007 ± 0.187 D, with 95% limits of agreement between −0.367 and +0.381); Galilei and WAM 5500, below (mean Δ ± SD: −0.148 ± 0.195 D, with 95% limits of agreement between −0.538 and +0.242).

**Figure 2:**
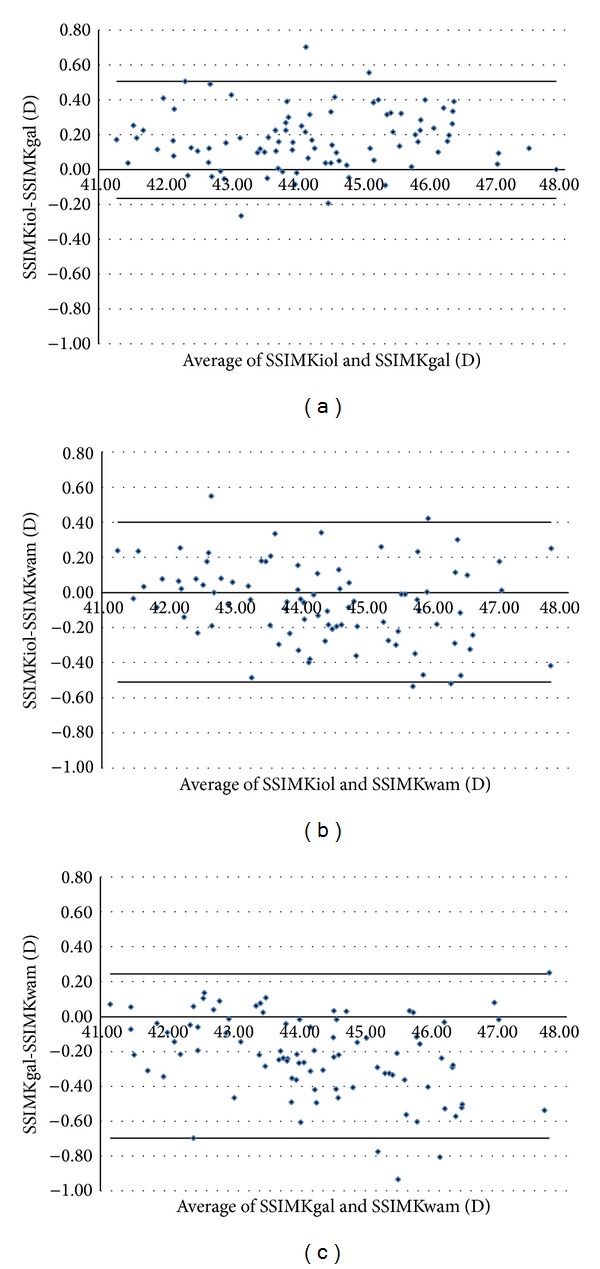
Bland-Altman analysis showing the distribution of the steep SimK differences on the *y*-axis and the average of the instrument readings on the *x*-axis. IOL Master and Galilei, above (mean Δ ± SD: +0.171 ± 0.168 D, with 95% limits of agreement between −0.165 and +0.507); IOL Master and WAM 5500, middle (mean Δ ± SD: −0.055 ± 0.227 D, with 95% limits of agreement between −0.509 and +0.399); Galilei and WAM 5500, below (mean Δ ± SD: −0.226 ± 0.236 D with 95% limits of agreement between −0.698 and +0.246).

**Figure 3:**
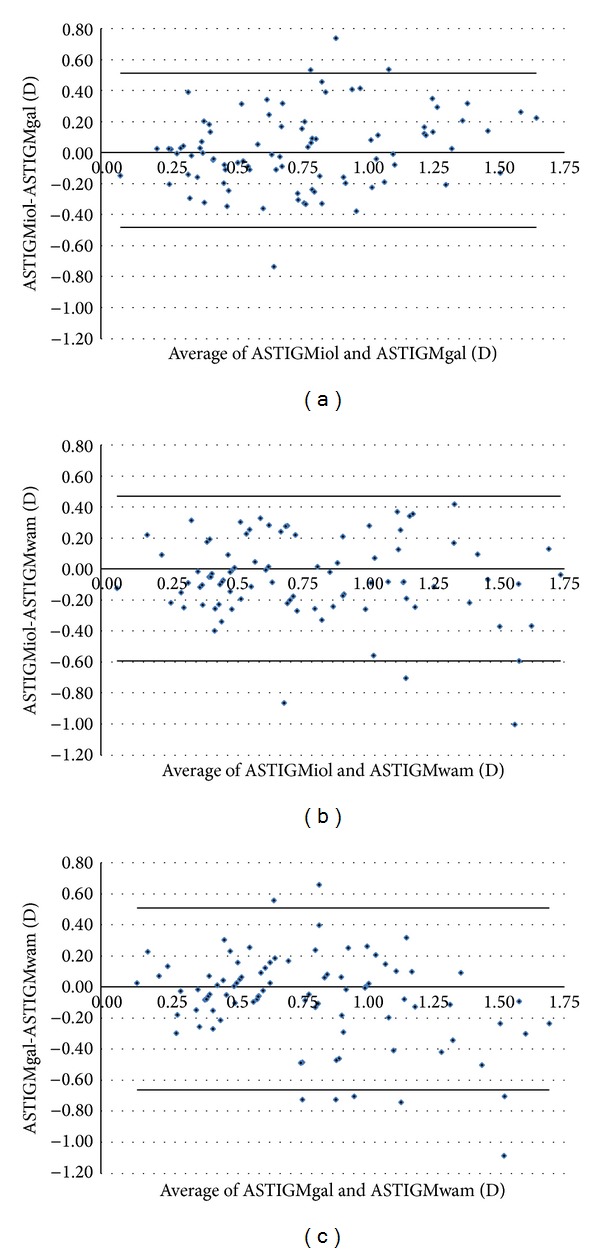
Bland-Altman analysis showing the distribution of the corneal astigmatism differences on the *y*-axis and the average of the instrument readings on the *x*-axis. IOL Master and Galilei, above (mean Δ ± SD: +0.016 ± 0.249 D, with 95% limits of agreement between −0.482 and +0.514); IOL Master and WAM 5500, middle (mean Δ ± SD: −0.061 ± 0.265 D, with 95% limits of agreement between −0.591 and +0.469); Galilei and WAM 5500, below (mean Δ ± SD: −0.078 ± 0.292 D, with 95% limits of agreement between −0.662 and +0.506).

**Figure 4:**
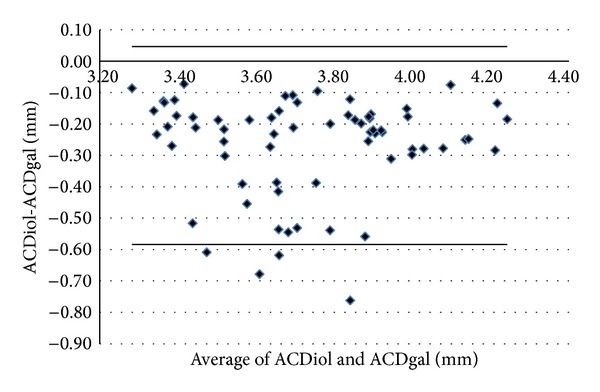
Bland-Altman analysis showing the distribution of the ACD differences (ACDiol-ACDgal) on the *y*-axis and the average of the instrument readings (ACDiol + ACDgal)/2 on the *x*-axis. Mean Δ ± SD: −0.269 ± 0.158 mm, with 95% limits of agreement between −0.584 and +0.047.

**Figure 5:**
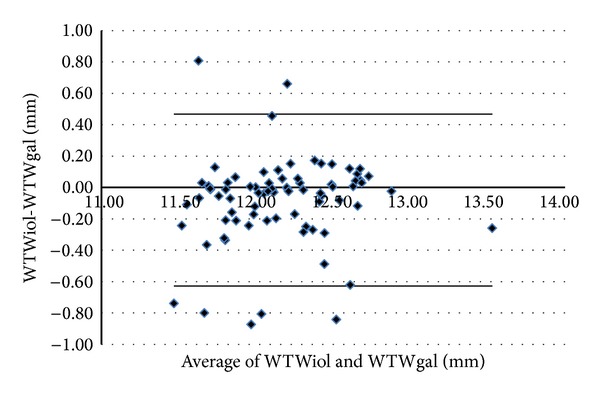
Bland-Altman analysis showing the distribution of the WTW differences (WTWiol-WTWgal) on the *y*-axis and the average of the instrument readings (WTWiol + WTWgal)/2 on the *x*-axis. Mean Δ ± SD: −0.080 ± 0.274 mm, with 95% limits of agreement between −0.628 and +0.467.

Figures 1–5 show the Bland-Altman plots of the flat SimK, steep SimK, corneal astigmatism, ACD, and WTW reproducibility between the methods. The Galilei method provided lower values of the flat SimK and steep SimK than did the other methods (Figures 1 and 2). The WAM 5500 method provided higher values of corneal astigmatism than did the IOL Master and Galilei (Figure 3).  Figure 4 shows that all the values of the ACD obtained with the Galilei were higher than the values obtained by the IOL Master. In the majority of cases, using the Galilei resulted in higher measurements of the WTW than did the IOL Master (Figure 5).

## 5. Discussion

The partial coherence interferometry or Scheimpflug cameras have become important tools in surgery planning because of the importance of knowing the values of the ocular structures [18]. Accurate and predictable biometric measurements are required to guarantee the success of various surgeries [19, 20]. Previous studies have measured the anterior segment in normal subjects and in subjects with ocular disease using the IOL Master, Galilei, and WAM 5500 [3, 5, 8, 21–28].

A study of repeatability and correlations between these devices are important to establish whether results could be used interchangeably in clinical practice. Our ICC measurements reached nearly perfect reliability, which was in accordance with previous studies on the Galilei [25, 26, 28–31] and the IOL Master [19, 32–34]. Sheppard and Davies [5] reported a mean intrasession repeatability of WAM 5500 of 0.09 D for the spherical component, 0.14 D for the cylindrical component, and 0.07 D and 0.06 D for the J0 and J45; we did not find any other reports regarding the repeatability of the WAM 5500 method. Our results, with those of Sheppard and Davies [5], demonstrate the high repeatability of this device although the correlations and the Bland-Altman plots show the mean differences between the methods, and some of the measurement points were located outside of the 95% limits of agreement in all the cases.

In our study, the Galilei provided flat SimK (43.37 D) and steep SimK (44.11 D) values similar to the results of Crawford et al. [30] (43.3 D and 44.5 D, resp.); however, these values were lower than the values found by Demir et al. [21] (43.63 D and 44.71 D, resp.), and Aramberri et al. [25] reported even lower values of 42.70 D and 43.67 D. Shirayama et al. [7] obtained statistically significant differences in the mean corneal power values between the IOL Master (43.92 D) and the Galilei (43.80 D). In our study, the magnitudes of these differences were similar; the flat SimK measurements were 0.155 ± 0.156 D, and the steep SimK measurements were 0.171 ± 0.168 D. The IOL Master measures were slightly higher than those of the Galilei. The results indicate that the keratometry provided by the WAM 5500 is clinically interchangeable with that of the IOL Master, although the WAM 5500 measurements are not interchangeable with those of the Galilei. Comparisons of the keratometry performed with the WAM 5500 with previous studies are not possible because, to our knowledge, the previous studies only focused on refraction. The differences could be because the keratometric corneal values measured with the WAM 5500 and the IOL Master correspond to the paracentral area (3 mm and 2.3 mm, resp.), whereas the Galilei assesses a greater area of the corneal surface (8 mm).

Our results indicated that the mean ACD measurements between the two devices were strongly correlated. There was no statistically significant difference in the mean ACD between the devices; however, the Galilei measurements were, on average, longer than those of the IOL Master (0.269 mm). In our study, 94% of the difference in the measurements lies within 95% of the LoA (range 0.631 mm); these limits could indicate important errors in the IOL calculations in cataract surgery. An error of 1 mm in an ACD value affects the postoperative refraction by approximately 1.0 D in a myopic eye, 1.5 D in an emmetropic eye, and up to 2.5 D in a hyperopic eye [35]. A keratometric error of 1 D affects postoperative refraction by approximately the identical amount [35], with an error of approximately 1.3 to 1.6 D in the IOL power calculation [36]. These results are relatively similar to the results of Patel and Pandit [24]; although their study found statistically significant differences between the devices, the differences were lower than our differences (0.12 ± 0.11 mm). Our results were in agreement with the previously published LoA [8, 24, 37], although our absolute values of the ACD were slightly higher than the values of the previous reports.

Our experience with the performance of these measurements indicates that the IOL Master sometimes stimulates accommodation, and more myopic eyes have shorter ACD measurements [21]. In these cases, the differences with the Galilei are higher and must be individually assessed.

The absolute values of the ACD_IOL_, 3.60 mm, were similar to the values previously reported by Woodmass and Rocha [23] (3.78 mm), Rosa et al. [13] (3.62 mm), and Crawford et al. [30] (3.56 mm); however, our values were higher than the values by Vogel et al. [19] (3.25 mm) and Rosa et al. [13] (3.22 mm). This difference could be because of the age of the studied population; in the presence of a cataract, which is more common in older individuals, the ACD is shorter.

Caution should be used when the two instruments are used interchangeably. Small, although significant, differences could exist between the IOL Master and the Galilei in cases in which the ACD is measured and accommodation is not controlled. The ACD in clinically normal eyes of young people is measured similarly by these devices; however, the CCT should be corrected for the measurements to be comparable. The validity and clinical utility in clinical practice for assessing the IOL power calculation in pathologic eyes should be studied.

Statistically significant differences inthe WTW or corneal diameter were measured by the IOL Master and the Galilei (slightly higher values are obtained by the Galilei than by the IOL Master); there were a worse correlation and a higher LoA (1.095 mm) between the measures. The absolute values are equivalent to the values described with the IOL Master by Ortiz et al. [38], 12.19 mm, and by Baumeister et al. [39], 12.02 mm. Salouti et al. [40] described WTW values of 12.01 mm with the Galilei; all of those values were compared with the Orbscan, which provides lower results than the Galilei or the IOL Master.

These results were obtained in healthy young people with good fixation and good collaboration. Factors including age, irregular corneas, refractive surgery, or dry eyes could limit the fixation and tear stability and adversely affect the examination [41]. Possible reasons for the discrepancies between the measurements with the different devices include differences in the measuring principles and alignment errors. Additionally, the different measurement principles using the tools analysed are relevant. Further studies are needed to investigate how these facts affect the results of the different devices.

We determined that the three devices had a nearly perfect correlation in measuring anterior segment parameters with greater variability for corneal astigmatism terms in healthy young persons. In these persons, the evaluated parameters had very good repeatability, and their limits of agreement showed excellent clinical results for these devices. In our research, we obtained equivalent measurements between the WAM and the IOL Master. We found that the Galilei and the IOL Master or the Galilei and the WAM do not produce comparable values in measures of the anterior segment structures; however, the differences between the mean measurements of the instruments should not be considered clinically significant.

## Figures and Tables

**Table 1:** Mean values and variance of the flat SimK, steep SimK, corneal astigmatism, ACD, and WTW measurements by the IOL Master, Galilei, and WAM 5500 methods and the intraclass coefficients for three repeated measurements.

		Mean	*S*_*w*_ (±)	ICC	Lower 95% CI	Upper 95% CI	COV (%)
IOL Master	Flat SimK (D)	43.53	1.49	0.999	0.999	0.999	3.43
	Steep SimK (D)	44.28	1.52	0.999	0.998	0.999	3.44
	Corneal astigmatism (D)	0.76	0.42	0.971	0.959	0.980	55.35
	ACD (mm)	3.60	0.30	0.974	0.965	0.982	8.23
	WTW (mm)	12.12	0.42	0.967	0.953	0.978	3.47

Galilei	Flat SimK (D)	43.37	1.48	0.997	0.995	0.998	3.42
	Steep SimK (D)	44.11	1.51	0.998	0.997	0.999	3.43
	Corneal astigmatism (D)	0.74	0.40	0.855	0.793	0.901	53.54
	ACD (mm)	3.87	0.42	0.937	0.908	0.958	3.42
	WTW (mm)	12.20	0.46	0.937	0.908	0.958	3.79

WAM 5500	Flat SimK (D)	43.52	1.49	0.992	0.988	0.995	3.44
	Steep SimK (D)	44.34	1.59	0.988	0.981	0.992	3.58
	Corneal astigmatism (D)	0.82	0.48	0.790	0.679	0.862	58.78

*S*_*w*_: within-subjects variance; ICC: intraclass coefficients; CI: confidence interval; COV: coefficient of variation.

**Table 2:** The mean differences, standard deviation, and Wilcoxon test (values of *P* < 0.05 were considered to be indicative of significant differences) of the flat SimK, steep SimK, corneal astigmatism, ACD, and WTW measurements by the IOL Master, Galilei, and WAM 5500.

		Mean difference	SD (±)	*P *
Flat SimK (D)	IOL Master-Galilei	0.155	0.156	<0.001
	IOL Master-WAM 5500	0.007	0.187	0.302
	Galilei-WAM 5500	−0.148	0.195	<0.001

Steep SimK (D)	IOL Master-Galilei	0.171	0.168	<0.001
	IOL Master-WAM 5500	−0.055	0.227	0.172
	Galilei-WAM 5500	−0.226	0.236	<0.001

Corneal astigmatism (D)	IOL Master-Galilei	0.016	0.249	0.662
	IOL Master-WAM 5500	−0.061	0.265	0.055
	Galilei-WAM 5500	−0.078	0.292	0.022

ACD (mm)	IOL Master-Galilei	−0.269	0.158	<0.001

WTW (mm)	IOL Master-Galilei	−0.080	0.274	0.009

**Table 3:** Spearman correlation coefficients between devices.

		Ro Spearman	*P *
Flat SimK (D)	IOL Master-Galilei	0.992	<0.001
	IOL Master-WAM 5500	0.990	<0.001
	Galilei-WAM 5500	0.986	<0.001

Steep SimK (D)	IOL Master-Galilei	0.991	<0.001
	IOL Master-WAM 5500	0.986	<0.001
	Galilei-WAM 5500	0.984	<0.001

Corneal astigmatism (D)	IOL Master-Galilei	0.777	<0.001
	IOL Master-WAM 5500	0.792	<0.001
	Galilei-WAM 5500	0.754	<0.001

ACD (mm)	IOL Master-Galilei	0.809	<0.001

WTW (mm)	IOL Master-Galilei	0.791	<0.001
